# PROTOCOL: Instruments for the evaluation of emotional intelligence in persons with hearing impairments: A scoping review

**DOI:** 10.1002/cl2.1261

**Published:** 2022-06-30

**Authors:** Petra Potmesilova, Milon Potmesil, Jana Mareckova

**Affiliations:** ^1^ Department of Christian Education, St. Cyril and Methodius Faculty of Theology Palacký University Olomouc Czechia; ^2^ Center of Evidence‐based Education & Arts Therapies: A Joanna Briggs Institute Affiliated Group, Faculty of Education, Institute of Special Education Studies Palacký University Olomouc Czechia; ^3^ Department of Anthropology and Health Education Palacký University Olomouc Czechia

## Abstract

**Objective:**

This scoping review aims to identify and describe the instruments used to evaluate emotional intelligence in deaf and hard of hearing (D/HH) persons.

**Introduction:**

Emotional intelligence is a person's ability to work with emotions in response to a particular situation. Deafness or a hard of hearing has a negative effect on functioning in the field of emotional intelligence and leads to a specific approach to the diagnosis or evaluation of emotional intelligence.

**Inclusion Criteria:**

The scoping review will consider studies focused on persons who are deaf or hard of hearing at any age. The review will consider studies that feature existing instruments and the process of the evaluation of emotional intelligence in people with hearing disabilities. Relevant information will include the type, extent, and form of possible modification of specific instruments and approaches. Criteria for the exclusion of the study will be in terms of the target category being persons with a cochlear implant, psychiatric diagnoses, persons who have become deaf, and persons with multiple disabilities.

**Methods:**

The review will be conducted following the JBI methodology for scoping reviews. The databases to be searched include MEDLINE, Embase, CINAHL Plus, ProQuest Central, PsycINFO, Scopus, Web of Science, Clinical Trials, and the Cochrane Central Register of Controlled Trials (CENTRAL), and Google Scholar. The search for unpublished studies will include ProQuest Dissertations and Theses (ProQuest). Eligible studies will undergo data extraction by two independent reviewers using a tool created by the authors. Along with a narrative summary, the results will be presented in diagrammatic or tabular form.

## 1 INTRODUCTION

### 1.1 Emotional intelligence

Two completely different models characterize the concept of emotional intelligence. The first model of emotional intelligence that will be included in the search strategy is the Ability model (Salovey et al., 2004). It is the concept of emotional intelligence, which is perceived as a set of interconnected and objectively measurable cognitive‐emotional abilities, including the ability to perceive, direct, facilitate and understand the emotions of oneself and others. The model contains four main types of emotional abilities. Emotional perception—refers to an individual's ability to recognize their own emotions and understand emotions expressed by others. Emotional use is the ability to use one's own emotions in thinking, making decisions, and conceiving relationships. Emotional understanding—refers to the processing of specific information that provides different emotions read from a person's behavior. The fourth ability is emotional management. Individuals can control their own emotions and respond to other people's emotions. The Trait Model is a model that represents the opposite concept of the Ability model (Petrides, 2021). It is a model of emotional intelligence based on individuals' ability to perceive their emotional abilities. The trait model of emotional intelligence is characterized as "a constellation of emotional self‐perceptions and is related to a person's personality. It is assumed that emotions are subjective and emotional intelligence is the individual perception of one's ability to work with emotions. Emotional intelligence is a person's ability to work with emotions in response to a particular situation and evaluate emotions to regulate behavior (Mayer & Salovey, 1997; VandenBos, 2015). Emotional intelligence consists of the ability to orientate oneself in one's own emotions, manage them, and subsequently make appropriate use of them in the conception and realization of social behavior. Emotional intelligence is a prerequisite for handling social situations and is closely linked to childhood experience and social learning (Basu & Mermillod, 2011; Boyatzis, 2018). The process of learning and regulating reactions in connection with emotions and their experience is closely connected with verbalization, i.e., the use of the individual's language and communication competencies to develop the full functionality of emotional intelligence (Alba‐Juez & Pérez‐González, 2019; Andrienko et al., 2020).

Emotional intelligence can be evaluated and measured. There are several tests of emotional intelligence; their results help determine not only its level but also the possibility of practical work with the client, focused on working with emotions and their control.

### 1.2 Testing emotional intelligence

We list the most commonly mentioned instruments used to measure emotional intelligence. The test name shows the number of occurrences in the Google search engine. Emotional & Social Competence Inventory—University Edition—96,600,000; Wong's Emotional Intelligence Scale (WEIS)—767,000; Group Emotional Competence (GEC) Inventory—740,000; The Emotional Quotient Inventory 2.0 (EQ‐i 2.0, EQ‐360)—404,000; The Genos Emotional Intelligence Inventory (Genos EI)—42,700; Trait Emotional Intelligence Questionnaire (TEIQue)—36,400; Emotional and Social Competence Inventory (ESCI)—33,900; Mayer‐Salovey‐Caruso EI Test (MSCEIT)—33,700; Geneva Emotional Competence Test (GECo)—27,000; The Schutte Self Report Emotional Intelligence Test (SSEIT)—23,900; Work Group Emotional Intelligence Profile (WEIP)—21,200. The information that all these tests are verbal can be considered essential (O'Connor et al., 2019). This fact means that testing is in all cases standardized for individuals with a typically developed ability to read with understanding. Therefore, the language competencies of the client or respondent are necessary for understanding the assignment or statement in the test to consider the answer valid.

### 1.3 Deafness and hard of hearing

Deafness or hard of hearing has a negative impact on one's development in the cognitive, social, and emotional areas from an early age (Overview of Psychological Testing, 2015). Hearing is the essential instrument for awareness of emotions and emotional experience in the process of social learning (Abdolrezapour & Tavakoli, 2021). The barrier of D/HH is the most common cause of misunderstandings of meaning and subtle differentiations in the range of emotions and reflections of emotional states following the evaluation of a particular social situation and subsequent manifestation of behavior. Everything is based on adequate control of concepts, language, and communication. The age of the child when hearing loss is diagnosed is an essential factor that will affect the initiation of rehabilitation and its effectiveness (Laugen et al., 2017; Marschark, 1997; Yasin et al., 2012). Any delayed diagnosis has a negative effect on deviations and delays from typical socio‐emotional and cognitive development. This fact is related to the delayed development of communication competencies, language, and speech. Ultimately, the child's educational level is also at risk. It should be stressed that communication and language are essential for a child's further ability to learn. Whether it is spoken language or sign language makes no difference (Lederberg & Spencer, 2009; Quer & Steinbach, 2019). The negative effect of deafness or severe hearing loss affects such a disabled person throughout his or her life to objectively diagnose emotional intelligence in people who are deaf or severely hard of hearing, to take into account specific language comprehension requirements, the subject of this study is to look for the tests used, modified conditions, and comparable test results with a control group of hearing respondents (Elliott et al., 2002). This scoping review will examine and gather evidence concerning the instruments used with persons who are deaf or hard of hearing, taking into account the psychometric parameters and their necessary adjustment. A preliminary search of MEDLINE, PsycInfo, Scopus, the Cochrane Database of Systematic Reviews, and JBI Evidence Synthesis was conducted. Sources of unpublished studies/gray literature to be searched include Google Scholar and Researchgate.

The following search terms were used: deaf, hard of hearing, emotional intelligence, test, testing.

The subject of the upcoming scoping review is to look for published primary research, registered protocols, and unindexed and gray literature that deal with the topic of emotional intelligence and its measurement in people who are deaf or severely hard of hearing.

## 2 REVIEW QUESTION

Which instruments or approaches are used in the evaluation of Emotional intelligence in D/HH persons?

## 3 KEYWORDS

deaf; emotional intelligence; evaluation; hard of hearing; test; testing.

## 4 INCLUSION CRITERIA

### 4.1 Participants

The scoping review will consider studies focused on persons who are deaf or hard of hearing. The age of the target group will not be a limiting factor when searching. The criteria for the exclusion of the study will be in terms of the target category of persons with a cochlear implant, persons with psychiatric diagnoses, persons who became deaf after 7 years of age, and persons with multiple disabilities according to the International Classification of Functioning, Disability, and Health (WHO, 2001).

### 4.2 Concept

The review will consider studies that feature existing instruments and the process of their use in the evaluation of emotional intelligence in people with hearing disabilities. Non‐standardized instruments and the unclear process of their use in the evaluation of emotional intelligence in people with hearing disabilities will be excluded.

### 4.3 Context

Relevant information will include the type, extent, and form of possible modification of specific instruments and approaches. These adjustments can be assumed because evaluation instruments and techniques are primarily designed and standardized for a healthy—hearing—population. Another essential objective of the review will be the information obtained on the necessary preparation of the experts who carry out the evaluation. It will most probably be psychologists or special pedagogues.

### 4.4 Types of sources

The scoping review will include qualitative and quantitative studies focused on the target group and emotional intelligence evaluation. The scoping review will consist of sought‐after studies, present primary research, randomized controlled trials, non‐randomized controlled trials, longitudinal studies, case studies, and systematic reviews. Gray literature and non‐indexed literature will also be included in the search. This review will also consider descriptive observational study designs including case series, individual case reports, and descriptive cross‐sectional studies for inclusion.

Qualitative studies will also be considered that focus on qualitative data, including, but not limited to, designs such as phenomenology, grounded theory, ethnography, qualitative description, action research, and feminist research.

In addition, systematic reviews that meet the inclusion criteria will also be considered, depending on the research question. Text and opinion papers will also be considered for inclusion in this scoping review.

## 5 METHODS

The proposed scoping review will be conducted following the JBI methodology for scoping reviews (Peters et al., 2020).

### 5.1 Search strategy

The search will be aimed at published and unpublished primary studies and non‐indexed and gray literature sources. Keywords for texts will be searched for in their title or abstract. Keywords in the research and abstract names were the basis for constructing a complete search strategy (Supporting Information: Annex 1). The search strategy will be formally adjusted to meet sensitive search requirements in a particular database. In terms of time limitation, the search will be limited to between 1963 and 2021. In 1963, a study (Horst, 1963) was published in which the author describes a program for testing high school students. Studies in English, German, and French will be included in the search.

### 5.2 Information sources

#### 5.2.1 Information resources

The MEDLINE, Embase, CINAHL Plus, ProQuest Central, PsycINFO, Scopus, Web of Science Core Collection, Clinical trials, Cochrane Central Register of Controlled Trials (CENTRAL), and Google Scholar databases will be used for the search. The methodology for effective search in the above‐listed databases will be according to the recommendations of Bramer et al. (2017).

### 5.3 Study of evidence selection

The citations that are identified will be centered and uploaded to EndNote 20/2021(Clarivate Analytics, PA, USA) and removed from all duplicates when searched. Two independent reviewers will review each article's titles and abstracts in terms of compliance with the inclusion and exclusion criteria. Full‐text articles with content related to the topic of the study will be collected and citations exported to the JBI System for the Unified Management, Assessment, and Review of Information (JBI SUMARI) (Munn et al., 2019). Two independent reviewers will rate the articles from the point of view of the inclusion criteria. All reasons for elimination will be stated in the scoping review. If the reviewers disagree, a third independent reviewer will be invited.

The search results and the study inclusion process will be reported in full in the final scoping review and presented in a Preferred Reporting Items for Systematic Reviews and Meta‐analyses extension for scoping review (PRISMA‐ScR) flow diagram (Tricco et al., 2018); studies that do not meet the requirements, such as short messages, conferences, and single abstracts, will be excluded from the study. All acquired sources of gray literature will be subjected to a strict quality assessment (quality dissertation vs. nonprofessional texts). Authors will use any systematic review to orient the topic links to search for primary sources. Method‐forward‐backward search will be used to ensure the correctness of the procedure.

### 5.4 Data extraction

The data will be obtained from articles included in the scoping review by two independent reviewers. The data collected will contain specific details about the target population, the instrument used, and the approaches to assessing emotional intelligence. If there is no consensus among the reviewers in the first level, a third independent reviewer will be invited for consultation. Any disagreements that arise between the reviewers (P. P., M. P.) will be resolved through discussion, or with an additional reviewer (J. M.). If necessary, the study's authors will contact the authors of specific articles to request that the required data be supplemented. When assessing the quality of the searched texts, the recommended procedure will be used according to Harrison et al. (2021).

### 5.5 Data analysis and presentation

The data obtained from selected articles will be presented in tabular and textual narrative form to provide an overview of the research question's details. In addition to summing up the results and answering the research question, options for further research activities will be proposed. The tabulated/diagrammatic results, together with a narrative summary, will describe how the results relate to the review question and objectives. The narrative meta‐synthesis of the processed studies will follow the procedure recommended by Popay et al. (2005).

## CONTRIBUTIONS OF AUTHORS

The authors confirm their contribution to the paper as follows: study conception and design: P. Potmesilova, M. Potmesil, J. Mareckova; data collection, analysis, and interpretation of results: P. Potmesilova, M. Potmesil, draft manuscript preparation: P. Potmesilova, M. Potmesil, J. Mareckova. All authors reviewed the results and approved the final version of the manuscript. The text has undergone a revision by a professional translator—a native English speaker S. Gill.

## CONFLICTS OF INTEREST

There is no conflict of interest in this project.

## Supporting information

Supporting information.

## References

[cl21261-bib-0001] Abdolrezapour, P. , & Tavakoli, M. (2021). The relationship between emotional intelligence and EFL learners' achievement in reading comprehension. Innovation in Language Learning and Teaching, 6(1), 1–13. 10.1080/17501229.2010.550686

[cl21261-bib-0002] Alba‐Juez, L. , & Pérez‐González, J.‐C. (2019). Emotion and language “at work”: The relationship between trait emotional intelligence and communicative competence as manifested at the workplace. In J. L. Mackenzie & L. Alba‐Juez (Eds.), Emotion in discourse (pp. 247–278). John Benjamins Publishing Company. 10.1075/pbns.302.10alb

[cl21261-bib-0003] Andrienko, T. , Chumak, N. , & Genin, V. (2020). Emotional intelligence and acquisition of English language oral communication skills. Advanced Education, 7(15), 66–73. 10.20535/2410-8286.201013

[cl21261-bib-0004] Basu, A. , & Mermillod, M. (2011). Emotional intelligence and social‐emotional learning: An overview. Online Submission, 1(3), 182–185.

[cl21261-bib-0005] Boyatzis, R. E. (2018). The behavioral level of emotional intelligence and its measurement. Frontiers in Psychology, 9, 1438. 10.3389/fpsyg.2018.01438 30150958 PMC6099111

[cl21261-bib-0006] Bramer, W. M. , Rethlefsen, M. L. , Kleijnen, J. , & Franco, O. H. (2017). Optimal database combinations for literature searches in systematic reviews: A prospective exploratory study. Systematic Reviews, 6(1), 1–12. 10.1186/s13643-017-0644-y 29208034 PMC5718002

[cl21261-bib-0007] Committee on Psychological Testing, Including Validity Testing, for Social Security Administration Disability Determinations; Board on the Health of Select Populations; Institute of Medicine . (2015). Psychological testing in the service of disability determination. National Academies Press (US). June 29. 3, Overview of Psychological Testing. https://www.ncbi.nlm.nih.gov/books/NBK305233/

[cl21261-bib-0008] Elliott, S. N. , McKevitt, B. C. , & Kettler, R. J. (2002). Testing accommodations research and decision making: the case of “good” scores being highly valued but difficult to achieve for all students. Measurement & Evaluation in Counseling & Development, 35(3), 153. 10.1080/07481756.2002.12069060

[cl21261-bib-0009] Harrison, R. , Jones, B. , Gardner, P. , & Lawton, R. (2021). Quality assessment with diverse studies (QuADS): An appraisal tool for methodological and reporting quality in systematic reviews of mixed‐ or multi‐method studies. BMC Health Services Research, 21(1), 144. 10.1186/s12913-021-06122-y 33588842 PMC7885606

[cl21261-bib-0010] Horst, P. (1963). The statewide testing program. Personnel & Guidance Journal, 41(5), 394–402.

[cl21261-bib-0011] Laugen, N. J. , Jacobsen, K. H. , Rieffe, C. , & Wichstrøm, L. (2017). Emotion understanding in preschool children with mild‐to‐severe hearing loss. Journal of Deaf Studies and Deaf Education, 22(2), 155–163. 10.1093/deafed/enw069 27881481 PMC5881266

[cl21261-bib-0012] Lederberg, A. R. , & Spencer, P. E. (2009). Word‐learning abilities in deaf and hard‐of‐hearing preschoolers: Effect of lexicon size and language modality. Journal of Deaf Studies and Deaf Education, 14(1), 44–62. 10.1093/deafed/enn021 18495655

[cl21261-bib-0013] Marschark, M. (1997). Psychological development of deaf children (p. 1997). Oxford University Press.

[cl21261-bib-0014] Mayer, J. D. , & Salovey, P. (1997). What is emotional intelligence? In P. Salovey & D. J. Sluyter (Eds.), Emotional development and emotional intelligence: Educational implications (pp. 3–34). Harper Collins.

[cl21261-bib-0015] Munn, Z. , Aromataris, E. , Tufanaru, C. , Stern, C. , Porritt, K. , Lockwood, C. , Stephenson, M. , Moola, S. , Lizarondo, L. , McArthur, A. , Peters, M. , Pearson, A. , Jordan, Z. , & Farrow, J. (2019). The development of software to support multiple systematic review types: The Joanna Briggs Institute System for the Unified Management, Assessment and Review of Information (JBI SUMARI). International Journal of Evidence‐Based Healthcare, 17(1), 36–43. 30239357 10.1097/XEB.0000000000000152

[cl21261-bib-0016] O'Connor, P. J. , Hill, A. , Kaya, M. , & Martin, B. (2019). The measurement of emotional intelligence: A critical review of the literature and recommendations for researchers and practitioners. Frontiers in Psychology, 10, 1116. 10.3389/fpsyg.2019.01116 31191383 PMC6546921

[cl21261-bib-0017] Peters, M. , Godfrey, C. , McInerney, P. , Munn, Z. , Trico, A. , & Khalil, H. (2020) Chapter 11: Scoping reviews. In E. Aromataris & Z. Munn (Eds.). JBI Manual for Evidence Synthesis. Retrieved [cited May 24, 2021]. Retrieved October 11, 2020, from https://synthesismanual.jbi.global

[cl21261-bib-0018] Petrides, K. V. (2021). Radix intelligence: A new definition and integrative model of intelligence. Personality and Individual Differences, 169 ​. 10.1016/j.paid.2019.109784

[cl21261-bib-0019] Popay, J. , Roberts, H. , Sowden, A. , Petticrew, M. , Britten, N. , Arai, L. , Roen, K. , & Rodgers, M. (2005). Developing guidance on the conduct of narrative synthesis in systematic reviews. Journal of Epidemiology and Community Health, 59(Suppl. 1), A7.

[cl21261-bib-0020] Quer, J. , & Steinbach, M. (2019). Handling sign language data: The impact of modality. Frontiers in Psychology, 10 ​. 10.3389/fpsyg.2019.00483 PMC6423168 30914998

[cl21261-bib-0021] Salovey, P. , Brackett, M. A. & Mayer, J. D. (Eds.). (2004). Emotional intelligence: Key readings on the Mayer and Salovey model. Dude Publishing.

[cl21261-bib-0022] Tricco, A. C. , Lillie, E. , Zarin, W. , O'brien, K. K. , Colquhoun, H. , Levac, D. , Moher, D. , Peters, M. , Horsley, T. , Weeks, L. , Hempel, S. , Akl, E. A. , Chang, C. , McGowan, J. , Stewart, L. , Hartling, L. , Aldcroft, A. , Wilson, M. G. , Garritty, C. , … Straus, S. E. (2018). PRISMA extension for scoping reviews (PRISMA‐ScR): Checklist and explanation. Annals of Internal Medicine, 169(7), 467–473. 30178033 10.7326/M18-0850

[cl21261-bib-0023] VandenBos, G. R. (Ed.). (2015). APA dictionary of psychology (2nd ed.). American Psychological Association. 10.1037/14646-000

[cl21261-bib-0024] World Health Organization . (2001). International classification of functioning, disability and health: ICF. https://apps.who.int/iris/handle/10665/42407

[cl21261-bib-0025] Yasin, M. H. M. , Bari, S. , & Salubin, R. (2012). Emotional intelligence among deaf and hard of hearing children. Social Sciences, 7(5), 679–682. 10.3923/sscience.2012.679.682

