# Supplementary material for: PROTOCOL: Instruments for the evaluation of emotional intelligence in persons with hearing impairments: A scoping review
Source: Campbell Syst Rev. 2022 Jun 30;18(3):e1261. doi: 10.1002/cl2.1261 (PMC9246291; doi:10.1002/cl2.1261)
Supplement: Supplementary file 1 — Supporting information. [file CL2-18-e1261-s001.docx]

Appendices

### Appendix I: Search strategy

MEDLINE(R) 1946

Search conducted on May 19, 2021

| Search | Query | Results |
| --- | --- | --- |
| 1 | “Emotions/” [Mesh] OR “emotion*” [Ti/Ab/Key] OR “emotional intelligence” [Ti/Ab/Key] OR “emotional understanding” [Ti/Ab/Key] OR “emotional regulation*” [Ti/Ab/Key] OR “emotional experience*” [Ti/Ab/Key] OR “feeling*” [Ti/Ab/Key] OR “regret*” [Ti/Ab/Key] | 244,662 |
| 2 | “Deafness/ OR Hearing Loss/” [Mesh] OR “deafness” [Ti/Ab/Key] OR “deaf” [Ti/Ab/Key] OR “hearing loss” [Ti/Ab/Key] OR “hearing impairment*” [Ti/Ab/Key] OR “hearing disabilit*” [Ti/Ab/Key] OR “hearing disorder*” [Ti/Ab/Key] OR “hypoacus?s” [Ti/Ab/Key] OR “hearing impaired” [Ti/Ab/Key] | 85,605 |
| 3 | “Emotional & Social Competence Inventory” [Ti/Ab/Key] OR “Wong's Emotional Intelligence Scale” [Ti/Ab/Key] OR “Group Emotional Competence” [Ti/Ab/Key] OR “The Emotional Quotient Inventory” [Ti/Ab/Key] OR “The Genos Emotional Intelligence Inventory” [Ti/Ab/Key] OR “Trait Emotional Intelligence Questionnaire” [Ti/Ab/Key] OR “Mayer-Salovey-Caruso EI Test” [Ti/Ab/Key] OR “Geneva Emotional Competence Test” [Ti/Ab/Key] “The Schutte Self Report Emotional Intelligence Test” [Ti/Ab/Key] OR “Work Group Emotional Intelligence Profile” [Ti/Ab/Key] OR “ESCI” [Ti/Ab/Key] OR “WEIP” [Ti/Ab/Key] OR “GECo” [Ti/Ab/Key] OR “SSEIT” [Ti/Ab/Key] OR “MSCEIT” [Ti/Ab/Key] OR “TEIQue” [Ti/Ab/Key] OR “GENOS Ei” [Ti/Ab/Key] OR “EQ-i 2.0” [Ti/Ab/Key] “EQ-360” [Ti/Ab/Key] OR “GEC” [Ti/Ab/Key] OR “WEIS” [Ti/Ab/Key] OR “scale*” [Ti/Ab/Key] OR “questionnaire*” [Ti/Ab/Key] OR “test*” [Ti/Ab/Key] OR “evaluation” [Ti/Ab/Key] | 4,068,735 |
| 4 | #1 AND #2 | 1,217 |
| 5 | #4 AND #3 | 566 |

PsycINFO

Search conducted on May 20, 2021

| Search | Query | Results |
| --- | --- | --- |
| 1 | “Emotions OR Emotional Intelligence” [MA MeSH Subject Heading] OR “emotion*” [Ti/Ab] OR “emotional intelligence” [Ti/Ab] OR “emotional understanding and expression” [Ti/Ab] OR “emotional regulation*” [Ti/Ab] OR “emotional experience*“ [Ti/Ab] OR “feeling*” [Ti/Ab] OR “regret*” [Ti/Ab] | 416,905 |
| 2 | “Deaf-Blind Disorder” [MA MeSH Subject Heading] OR “deafness” [Ti/Ab] OR “deaf” [Ti/Ab] OR “hearing loss” [Ti/Ab] OR “hearing impair*” [Ti/Ab] OR “hearing disabilit*” [Ti/Ab] OR “hearing disorder*” [Ti/Ab] OR “hard of hearing” [Ti/Ab] OR “hypoacus?s” [Ti/Ab] | 27,638 |
| 3 | “Emotional & Social Competence Inventory” [Ti/Ab] OR “Wongʼs Emotional Intelligence Scale” [Ti/Ab] OR “Group Emotional Competence” [Ti/Ab] OR “The Emotional Quotient Inventory” [Ti/Ab] OR “The Genos Emotional Intelligence Inventory” [Ti/Ab] OR “Trait Emotional Intelligence Questionnaire” [Ti/Ab] OR “Mayer-Salovey-Caruso EI Test” [Ti/Ab] OR “Geneva Emotional Competence Test” [Ti/Ab] “The Schutte Self Report Emotional Intelligence Test” [Ti/Ab] OR “Work Group Emotional Intelligence Profile” [Ti/Ab] OR “ESCI” [Ti/Ab] OR “WEIS” [Ti/Ab] OR “GECo” [Ti/Ab] OR “SSEIT” [Ti/Ab] OR “MSCEIT” [Ti/Ab] OR “TEIQue” [Ti/Ab] OR “GENOS Ei” [Ti/Ab] OR “EQ-i 2.0” [Ti/Ab] “EQ-360” [Ti/Ab] OR “GEC” [Ti/Ab] OR “WEIP” [Ti/Ab] OR “scale*” [Ti/Ab] OR “questionnaire*” [Ti/Ab] OR “test*” [Ti/Ab] OR “evaluation” [Ti/Ab] | 1,419,385 |
| 4 | #1 AND #2 | 1,623 |
| 5 | #3 AND #4 | 525 |

### Appendix II: Data extraction

| **Scoping review details** | |
| --- | --- |
| Scoping review title |  |
| Review objective |  |
| Review question |  |
| **Inclusion criteria** |  |
| (P) – persons who are deaf or hard of hearing;  the age of the target group will not be a limiting factor when searching |  |
| (C) – existing standardized instruments and the process of their use in the evaluation of emotional intelligence in people with hearing disabilities |  |
|  |  |
| **Exclusion criteria** |  |
| (P) – persons with a cochlear implant, persons with psychiatric diagnoses, persons who became deaf after seven years of age, the persons with multiple disabilities according to the ICF |  |
| (C) – non-standardized instruments and the unclear process of their use in the evaluation of emotional intelligence in people with hearing disabilities |  |
|  |  |
| **Evidence source details and characteristics** | |
| Citation details |  |
| Country |  |
| Context |  |
| Participants – details |  |
| **Results extracted from the source of evidence (concerning the concept of the scoping review)** | |
| What type of assessment instrument is mostly used for the evaluation of emotional intelligence in persons with deafness? |  |
| What modifications were used because of limited language competencies? |  |
| Is there any age or sex differences in the group of respondents? |  |
